# Osteopontin splice variants are differential predictors of breast cancer treatment responses

**DOI:** 10.1186/s12885-016-2484-x

**Published:** 2016-07-11

**Authors:** Krzysztof Zduniak, Anil Agrawal, Siddarth Agrawal, Md Monir Hossain, Piotr Ziolkowski, Georg F. Weber

**Affiliations:** Department of Pathology, Wroclaw Medical University, Wroclaw, Poland; Department of General and Oncological Surgery, Wroclaw Medical University, Wroclaw, Poland; Division of Biostatistics and Epidemilogy, Cincinnati Children’s Hospital Medical Center, Cincinnati, OH USA; University of Cincinnati Academic Health Center, College of Pharmacy, 3225 Eden Avenue, Cincinnati, OH 45267-0004 USA

**Keywords:** Tumor progression marker, Immunohistochemistry, Breast cancer, Chemotherapy, Hormone therapy, Radiation therapy

## Abstract

**Background:**

Osteopontin is a marker for breast cancer progression, which in previous studies has also been associated with resistance to certain anti-cancer therapies. It is not known which splice variants may mediate treatment resistance.

**Methods:**

Here we analyze the association of osteopontin variant expression before treatment, differentiated according to immunohistochemistry with antibodies to exon 4 and to the osteopontin-c splice junction respectively, with the ensuing therapy responses in 119 Polish breast cancer patients who presented between 1995 and 2008.

**Results:**

We found from Cox hazard models, logrank test and Wilcoxon test that osteopontin exon 4 was associated with a favorable response to tamoxifen, but a poor response to chemotherapy with CMF (cyclophosphamide, methotrexate, fluorouracil). Osteopontin-c is prognostic, but falls short of being a significant predictor for sensitivity to treatment.

**Conclusions:**

The addition of osteopontin splice variant immunohistochemistry to standard pathology work-ups has the potential to aid decision making in breast cancer treatment.

## Background

Biomarkers are important for guiding the diagnosis and treatment of cancer. Two broad groups comprise prognostic markers and predictive markers. Prognostic markers allow forecasts regarding the natural course of the disease. They differentiate between patients likely to have a good versus a poor outcome. By contrast, predictive markers provide upfront information regarding how likely a patient is to benefit from a specific treatment, and hence may guide the choice from available therapies. Anticipating treatment response or risk of treatment resistance is a critical need in cancer care. Relevant predictive markers mostly belong to the groups of drug targets, molecules associated with drug transport or metabolism, and regulators of apoptosis or DNA repair. As such, they are mechanistically involved in the drug response. In addition, because highly aggressive tumors are generally more difficult to manage than less aggressive ones, some prognostic indicators may also have predictive properties.

In the histopathologic assessment of breast cancer, the standard markers ER, PR, and HER2 identify drug targets, as ER-positive tumors are candidates for anti-estrogen treatment whereas HER2-positive tumors are candidates for treatment with trastuzumab. Further, the absence of all three marker molecules defines triple-negative breast cancers, which have a poor prognosis and limited treatment options. There is a lack of more refined predictive markers for treatment success in the disease.

In breast cancer, osteopontin is a biomarker for aggressiveness and for prognosis. Further, it has been described as a marker for treatment responses. Osteopontin causes breast cancer resistance to cyclophosphamide [1], doxorubicin [2–4], paclitaxel [4] and cisplatin [4] through its anti-apoptotic properties or through the upregulation of drug exporters. Its levels also are an indicator for progression under anastrozole [5]. According to two studies in a breast cancer model cell line, the suppression of osteopontin gene expression can enhance radiosensitivity and affect cell apoptosis, suggesting that the molecule may be a target for the improvement of radiotherapy [6, 7]. In all these cases, pan-osteopontin was measured. Osteopontin is subject to alternative splicing in cancer, and it is not known which splice form is responsible for conveying resistance to which specific treatment. The variant forms are distinguishable by antibodies to exon 4, recognizing osteopontin-a and osteopontin-b, or to the splice junction of osteopontin-c respectively. Here we test the association of osteopontin splice variants, expressed in the growths at the onset of cancer therapy, with the ensuing response to specific treatments.

## Methods

### Patients

This study contained 119 patients from Poland who presented between 1995 and 2008 (allowing the assessment of 5-year survival). All cases refer to invasive ductal carcinoma, grades 1, 2 and 3, with subtypes including few mucinous and tubular carcinomas. Information about the patients was received from the Department of General and Oncological Surgery, Wroclaw and from the Division of Oncological Surgery, Walbrzych, Poland. The inclusion criteria were size of tumor not larger than 50 mm, and no adjuvant chemotherapy at the time of immunohistochemistry. For all patients, who met these criteria, paraffin blocks were available for evaluation. The data comprised also information about pathological TNM (pTNM), BRCA1 status, HER2, ER and PR status, and family history (other cases of invasive breast carcinoma in the family). Ensuing treatment constituted combinations of 1. hormone therapy with tamoxifen; 2. chemotherapy with CMF (cyclophosphamide, methotrexate, fluorouracil) 6 courses every 28 days; 3. chemotherapy with AC (cyclophosphamide, doxorubicin) 4 courses every 21 days plus CMF 6 courses every 28 days; 4. radiotherapy to the chest (50 Gy; Mon-Fri 2 Gy) 5. radiotherapy to chest and axilla (50 Gy; Mon-Fri 2 Gy).

### Immunohistochemistry

For each antibody a formalin-fixed and paraffin-embedded biopsy specimen from cancer tissue was cut on a microtome in 5 μm slices. The antibodies used in this study, after blocking in 2 % donkey serum, were anti-hOPNc IgY (Gallus Immunotech), and LF161 (Larry Fisher). The IgY antibody recognizes the osteopontin-c splice junction and detects the molecule in immunohistochemistry. It was diluted 1:500 to 1:700. The polyclonal rabbit antibody LF161 for staining selectively exon 4 (present in osteopontin-a and -b) was used at 1:1000. For each antibody, the tissues were scored for intensity (maximum intensity of the sample 0, 1, 2, or 3) and percent positivity (0, 1, 2, or 3), separately for nuclei and cytoplasm. The intensity of staining in immunohistochemistry was evaluated as previously described [8] and the classification criteria of intensity followed a published source [9]. The score was given as 0 points if no staining was observed, 1 for weak, 2 for moderate and 3 points for strong staining. Points were assigned to each case by two pathologists who independently evaluated all microscopic slides and in the rare cases of discrepant initial scores, a final score was agreed on after discussion [8].

### Statistics

All statistical analyses were performed using SAS (North Carolina, USA). Correlations between osteopontin-c and clinicopathological variables were assessed with Pearson’s correlation test. Correlation coefficients of 0.1 to 0.3 are considered weak, 0.4-0.6 is moderate, and 0.7-0.9 is strong correlation. A *p*-value of 0.05 or lower indicates statistical significance. The primary methods for addressing the study purposes were Cox hazard models, logistic regression models, and the nonparametric Wilcoxon test. Odds ratios estimate the odds of death for a one-unit increase in the independent variable. Unadjusted odds ratios and 95 % confidence intervals were calculated to investigate the effects of the components of pathological scores on the odds of death. For survival under hormone therapy or chemotherapy, the biomarkers osteopontin-exon-4 and osteopontin-c were also analyzed in a multiple regression framework to adjust the effect for other covariates. Each model contained either osteopontin-exon-4 or osteopontin-c and each other biomarker (tumor size, lymph node involvement, grade, HER2, Progesterone Receptor, Estrogen Receptor, or BRCA1), added one-at-a-time. Cox hazard ratios, *p*-values, and Aikaike Information Criterion (AIC) were used.

## Results

### Patient characteristics

Of 119 patients, 46 women (39 %) died from breast cancer within 5 years while 73 women (61 %) were alive after this observation period. The average age at the time of immunohistochemistry was 53 years for non-survivors and 53 years for survivors. All patients, comprising both subgroups, underwent surgery consisting of either modified radical mastectomy with axillary dissection, or conservative breast surgery with axillary lymph node dissection and post-operative adjuvant therapy (Table 1).

**Table 1:** Patient characteristics

		n	%
T	0	1	0.8
	1	59	49.6
	2	33	27.7
	3	6	5.0
	undefined	20	16.8
N	0	58	48.7
	1	14	11.8
	2	16	13.4
	3	11	9.2
	undefined	20	16.8
grade	1	36	30.3
	2	65	54.6
	3	18	15.1
Her2	low	69	58.0
	high	29	24.4
	undefined	21	17.6
PR	-	64	53.8
	+	54	45.4
	undefined	1	0.8
ER	-	59	49.6
	+	59	49.6
	undefined	1	0.8
BRCA-1	wild type	52	43.7
	mutant	26	21.8
	undefined	41	34.5
familial	no	44	37.0
	yes	40	33.6
	undefined	35	29.4
chemotherapy	AC 4 courses every 21 days, CMF 6 courses every 28 days	34	28.6
	CMF 6 courses every 28 days	55	46.2
	no	30	25.2
radiation therapy	chest (50 Gy; Mon-Fri 2 Gy)	41	34.5
	chest/axilla (50 Gy; Mon-Fri 2 Gy)	31	26.1
	no	47	39.5
hormone treatment	no	54	45.4
	tamoxifen	62	52.1

The patient populations are described according to diverse clinical variables. *CMF* cyclophosphamide, methotrexate, fluorouracil, *AC* cyclophosphamide, doxorubicin

### Immunohistochemistry

The anti-Osteopontin-exon-4 antibody, which recognizes osteopontin-a and -b, stained selectively the cytoplasm. Most tumors displayed osteopontin-c predominantly in their nuclei. The markers correlated moderately between each other (OPNc nuclear intensity, OPNc nuclear percent positivity, exon 4 cytoplasmic intensity, exon 4 cytoplasmic percent positivity), but in contrast to earlier studies they did not correlate with grade (Table 2). Analysis for the association with survival by the markers under investigation (osteopontin-c, exon 4, tumor grade) reflected them as prognostic for outcome. In addition to analyzing the indicators in their original scale, we dichotomized the immunohistochemical biomarkers into low (0–1) or high (2–3). Only the logrank test for dichotomized osteopontin-c fell short of corroborating significance (Table 3).

**Table 2:** Marker correlations

		Exon 4 cyt.per.	Exon 4 cyt.int.	OPNc nucl.per.	OPNc nucl.int.	Tumor grade
exon 4	Pearson Correlation	1	**0.69115**	**0.41194**	**0.53769**	0.10199
cyt.per.	*p*-value		<0.0001	<0.0001	<0.0001	0.2697
exon 4	Pearson Correlation		1	0.31522	**0.50491**	0.21883
cyt.int.	*p*-value			0.0005	<0.0001	*0.0168*
OPNc	Pearson Correlation			1	**0.6636**	0.0679
nucl.per.	*p*-value				<0.0001	0.4631
OPNc	Pearson Correlation				1	0.08674
nucl.int.	*p*-value					0.3483
tumor grade	Pearson Correlation					1

The table shows Pearson correlation coefficients and *p*-values for pairwise comparison of the histopathologic markers (osteopontin-c staining intensity, osteopontin-c percent positivity, exon 4 staining intensity, exon 4 percent positivity) and tumor grade. Statistical significance is indicated by underlining, moderate correlation is shown in bold

**Table 3:** Marker correlations

	Parametric (lognormal)		Logrank		Wilcoxon	
	χ^2^	*p*-value	χ^2^	*p*-value	χ^2^	*p*-value
exon 4 intensity	4.82	0.0281	9.8692	0.0197	16.3818	0.0009
exon 4 high/low			7.9144	0.0049	15.5494	<0.0001
OPNc intensity	7.24	<0.0001	16.9014	0.0007	27.5348	<0.0001
OPNc high/low			2.1234	0.1438	6.4847	0.0109
tumor grade	7.63	0.0057	11.392	0.0098	9.5087	0.0232

Quantitative multivariable analysis and non-parametric tests for the prediction of survival by the markers under study (osteopontin-c, exon 4, tumor grade). Used were either the staining levels 0,1,2,3 (intensity) or the combination of 2 and 3 versus 0 and 1 (high/low) under various model assumptions. Underlined *p*-values are considered significant (*p* < 0.05)

### Cancer treatment

The patients were subjected to various combinations of hormone treatment, chemotherapy, and radiation. Hormone treatment was tamoxifen. Chemotherapy comprised one of two regimens, CMF (cyclophosphamide, methotrexate, fluorouracil 6 courses every 28 days) or AC/CMF (cyclophosphamide, doxorubicin, 4 courses every 21 days plus CMF 6 courses every 28 days). Radiotherapy was given either to the chest (50 Gy; Mon-Fri 2 Gy) or to chest and axilla (50 Gy; Mon-Fri 2 Gy). Except for hormone therapy, survival after treatment (chemotherapy yes/no, radiation therapy yes/no) was shorter than survival without treatment. This reflects that more comprehensive therapy was given to patients with more aggressive cancers, which are inherently associated with poor prognoses for survival (Table 4).

**Table 4:** Survival under treatment

	Years of survival		
Treatment	Mean	std	n
hormone no (all subgroups)	6.48	3.96	54
hormone yes (all subgroups)	7.44	4.35	64
hormone alone	9.89	2.00	18
hormone and chemo	6.63	4.38	16
hormone and radiation	5.10	4.63	10
hormone, chemo, radiation	7.10	4.84	21
chemo no (all subgroups)	8.30	3.79	30
chemo yes (all subgroups)	6.57	4.22	89
chemo alone	7.92	4.57	13
chemo and hormone	6.63	4.38	16
chemo and radiation	5.82	3.66	39
chemo, hormone, radiation	7.10	4.84	21
chemo (CMF 6 courses every 28 days)	6.27	4.36	55
chemo (AC 4 courses every 21 days, CMF 6 courses every 28 days)	7.06	4.00	34
radiation no (all subgroups)	8.23	3.90	47
radiation yes (all subgroups)	6.21	4.17	72
radiotherapy alone	10.00	1.41	2
radiation and hormone	5.10	4.63	10
radiation and chemo	5.82	3.66	39
radiation, chemo, hormone	7.10	4.84	21

Shown are mean survival times in years (mean), standard deviation (std), and number of cases (n) consecutive to the various combinations of treatment. When censored for 13-year survivors (who may be alive beyond 13 years), the mean survival of patients under hormone treatment is 6.423 years with standard error 0.5274, compared to survival under no hormone treatment of 7.262 years and standard error 0.5077

### Osteopontin variants and specific treatment regimens

**Fig. 1:**
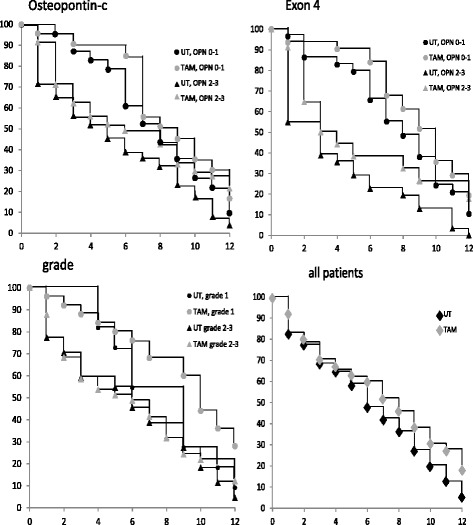
Kaplan-Meier survival curves for patients undergoing hormone therapy. Survival of patients under hormone treatment, distinguished according to low (levels 0–1) versus high (levels 2–3) osteopontin-c staining (top left), low versus high osteopontin-exon 4 staining (top right), or low (1) versus high (2–3) tumor grade (bottom left). The overall survival of patients receiving hormone treatment versus not receiving hormone treatment is shown for comparison on the bottom right. Untreated subgroups are displayed in black, treated subgroups in gray, low marker levels are shown as circles, high marker levels as triangles. The x-axis indicates years since diagnosis, the y-axis reflects % surviving patients

Tamoxifen is a selective estrogen receptor modulator that is used for the treatment of both early and advanced ER+ (estrogen receptor positive) breast cancers in pre- and post-menopausal women. Kaplan-Meier curves (Fig. 1) suggested a moderate survival benefit from treatment. When comparing hormone-treated to non-hormone-treated patients, low-grade cancers (grade 1) responded better to treatment than high-grade cancers (grade 2–3). In contrast, patients with high intensity staining (2–3) of exon 4 or osteopontin-c responded better to hormone therapy than those with low intensity staining (0–1) of these markers, as judged by a divergence with time between the hormone-treated and the non-hormone-treated patient groups at high marker intensity, but much less at low marker intensity.

**Fig. 2:**
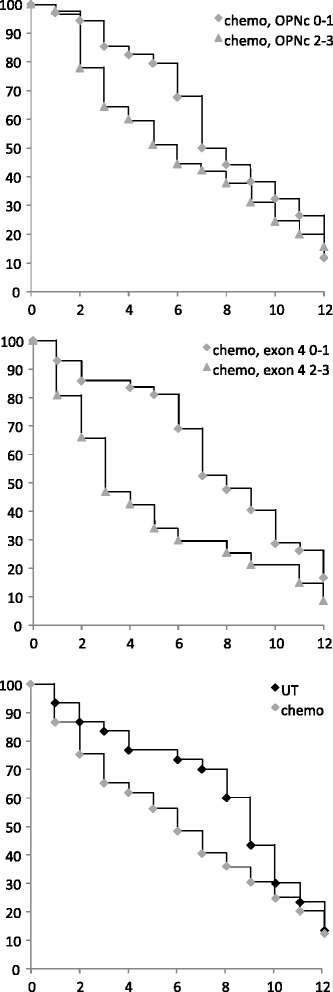
Kaplan-Meier survival curves for patients undergoing chemotherapy. Survival of patients under chemotherapy, distinguished according to low (0–1, diamonds) versus high (2–3, triangles) immunohistochemical markers. Shown are Kaplan Meier curves for osteopontin-c (top panel) or exon 4 (middle panel). For comparison, the survival of all patients treated (gray markers) or not treated (black markers) with chemotherapy is displayed (bottom panel). The x-axis indicates years since diagnosis, the y-axis reflects % surviving patients

For the analysis of chemotherapy responses, the comparison to the non-chemotherapy treated group was not meaningful, because the survival of the treated group was lower, which is not reflecting harm caused by the treatment but indicates the circumstance that chemotherapy was given to patients with aggressive tumors and poor prognoses. The survival curves of patients undergoing chemotherapy (Fig. 2), when distinguished according to low (0–1) versus high (2–3) immunohistochemical markers, confirm the poor survival prognosis associated with exon 4 and osteopontin-c [8], particularly reflected in a higher rate of patient deaths between 2 and 6 years after diagnosis in the high intensity group of each marker. Of note, the survival difference between exon 4 high and low appeared to be larger than between osteopontin-c high and low, implying the possibility that the marker could also be predictive of a poor chemotherapy response.

**Fig. 3:**
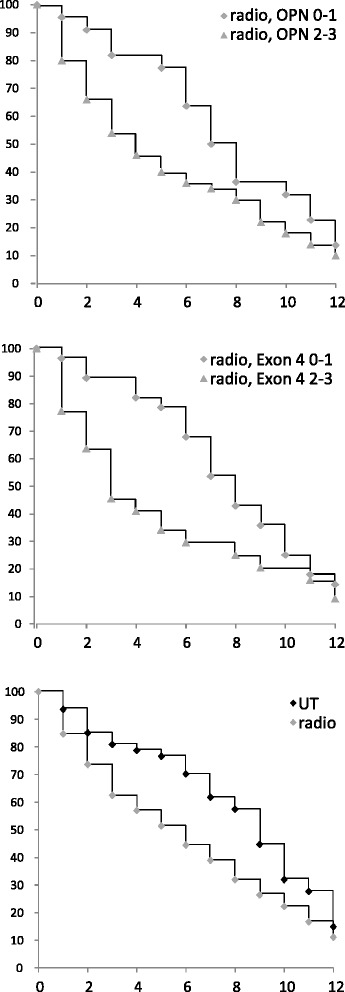
Kaplan-Meier survival curves for patients undergoing radiotherapy. Survival of patients under radiotherapy, distinguished according to low (0–1, diamonds) versus high (2–3, triangles) immunohistochemical markers. Shown are Kaplan Meier curves for osteopontin-c (top panel) or exon 4 (middle panel). For comparison, the survival of all patients receiving radiation (gray markers) or not treated with radiation (black markers) is displayed (bottom panel). The x-axis indicates years since diagnosis, the y-axis reflects % surviving patients

The Kaplan Meier survival curves of patients undergoing radiation treatment, distinguished according to low (0–1) versus high (2–3) immunohistochemical markers, again confirm the poor survival associated with exon 4 and osteopontin-c. Although the curves converge after about 10 years, during 2–6 years substantially more patients die in the high marker intensity groups than in the low marker intensity groups (Fig. 3).

### Osteopontin variants as predictive markers

The above findings (reduced survival of high versus low exon 4 and osteopontin-c under chemotherapy or radiation therapy) can be interpreted as poor therapy responses only if they are a) significant and b) distinct from the overall prognostic nature of these markers, as previously reported [8, 10, 11]. The seemingly favorable survival of high versus low exon 4 and osteopontin-c under hormone therapy is distinct from the prognostic characteristic, but requires testing for significance [12].

Cox hazard ratios (Table 5) showed that the staining intensity for osteopontin exon 4 was negatively correlated with survival in the non-hormone treated group, but not in the hormone treated group. This is consistent with a favorable response to tamoxifen associated with the presence of osteopontin exon 4. By contrast, osteopontin exon 4 was associated with a poor response to chemotherapy according to reduced survival by high level expressors in the treated group, but not in the non-chemotherapy treated group. CMF (not AC/CMF) was the regimen, the efficacy of which was compromised by high osteopontin exon 4. Because a supremum test indicated low confidence in the proportionality assumption, we sought to independently corroborate the analysis using the logrank and Wilcoxon tests (Table 6). According to all 3 tests, exon 4 is predictive of resistance to chemotherapy with CMF (as judged by significantly worse survival of the high marker intensity group), but not with AC/CMF. According to Cox hazard ratios and logrank test, exon 4 is a predictor of a favorable response to hormone therapy (the significantly worse survival associated with high marker intensity in the non-hormone treated group is lost under hormone therapy with tamoxifen). Although a similar predictive potential (for a favorable response to hormone therapy) is implied for osteopontin-c, the *p*-values for the Cox and logrank tests in the “hormone no” group fall just short of statistical significance.

**Table 5:** Survival under specific treatments

		Exon 4 intensity				Exon 4 %				OPN-c intensity				OPN-c %			
Treatment	n	Odds ratio	95 % CI	*p*-value	Supre-mum test	Odds ratio	95 % CI	*p*-value	Supre-mum test	Odds ratio	95 % CI	*p*-value	Supre-mum test	Odds ratio	95 % CI	*p*-value	Supre-mum test
hormone no (all subgroups)	54	0.503	**0.287-0.881**	0.016	0.104	1.087	0.624-1.895	0.767	0.908	0.620	0.353-1.089	0.096	0.178	1.210	0.696-2.102	0.500	0.954
hormone yes (all subgroups)	65	0.657	0.381-1.134	0.131	*<.0001*	0.687	0.391-1.206	0.191	0.056	0.826	0.463-1.473	0.517	*0.005*	1.101	0.630-1.923	0.735	0.704
hormone alone	18	1.534	0.430-5.467	0.509	0.614	1.122	0.420-2.998	0.819	0.616	1.661	0.607-4.542	0.323	0.518	1.113	0.383-3.235	0.845	0.607
hormone and chemo	16	0.412	0.129-1.316	0.134	*0.022*	0.637	0.173-2.343	0.497	0.064	0.634	0.204-1.973	0.432	*0.049*	1.352	0.441-4.148	0.598	0.269
hormone and radiation	10	0.358	0.069-1.862	0.222	0.180	0.495	0.096-2.558	0.401	0.568	0.250	0.029-2.180	0.209	0.185	0.827	0.192-3.561	0.798	0.597
hormone, chemo, radiation	21	0.598	0.169-2.112	0.424	0.763	0.604	0.218-1.676	0.333	0.801	0.867	0.278-2.710	0.806	0.375	0.894	0.310-2.580	0.836	0.810
chemo no (all subgroups)	30	0.711	0.320-1.580	0.403	*0.025*	0.794	0.366-1.722	0.560	0.233	0.903	0.391-2.084	0.811	0.134	0.978	0.425-2.254	0.959	0.612
chemo yes (all subgroups)	89	0.582	**0.371-0.913**	0.019	*0.001*	0.873	0.552-1.383	0.564	0.520	0.694	0.439-1.098	0.119	*0.003*	1.181	0.754-1.848	0.468	0.327
chemo (CMF)	55	0.524	**0.290- 0.943**	0.031	*0.008*	0.727	0.403-1.314	0.291	0.693	0.706	0.391-1.276	0.249	*0.013*	1.113	0.626-1.979	0.715	0.337
chemo (AC/CMF)	34	0.688	0.338-1.400	0.302	0.144	1.252	0.596-2.632	0.553	0.543	0.681	0.329-1.410	0.301	0.121	1.280	0.608-2.694	0.515	0.727
chemo alone	13	0.418	0.111-1.574	0.197	0.763	1.207	0.367-3.971	0.757	0.637	0.711	0.205-2.468	0.592	*0.047*	1.200	0.362-3.980	0.766	0.560
chemo and radiation	39	0.546	0.285-1.047	0.069	*0.027*	1.343	0.686-2.629	0.389	0.449	0.623	0.320-1.213	0.164	0.444	1.215	0.641-2.304	0.551	0.950
radiation no (all subgroups)	47	0.647	0.333-1.259	0.200	*0.024*	0.973	0.519-1.824	0.933	0.203	0.981	0.527-1.826	0.951	0.105	1.400	0.746-2.627	0.294	0.404
radiation yes (all subgroups)	72	0.626	0.376-1.044	0.073	*0.002*	0.774	0.466-1.285	0.321	0.489	0.675	0.394-1.157	0.153	*0.036*	1.086	0.659-1.791	0.745	0.832

Cox hazard ratios. The immunohistochemistry results for osteopontin-c and exon 4 were categorized into high staining (path scores 2 and 3) or low staining (path scores 0 and 1). Significant *p*-values are underlined, and confidence intervals that do not contain 1.0 are shown in bold. The last column displays the *p*-value for the supremum test, which assesses the proportionality assumption. *p*-values in italics are significant and indicate low confidence in the proportionality

**Table 6:** Survival under specific treatments

		Exon 4 intensity				Exon 4 %				OPN-c intensity				OPN-c %			
		Logrank		Wilcoxon		Logrank		Wilcoxon		Logrank		Wilcoxon		Logrank		Wilcoxon	
Treatment	n	χ^2^	*p*-value	χ^2^	*p*-value	χ^2^	*p*-value	χ^2^	*p*-value	χ^2^	*p*-value	χ^2^	*p*-value	χ^2^	*p*-value	χ^2^	*p*-value
hormone no (all subgroups)	54	7.127	0.008	9.087	0.003	0.106	0.745	0.015	0.902	3.384	0.066	5.373	0.021	0.551	0.458	0.372	0.542
hormone yes (all subgroups)	65	2.631	0.105	6.981	0.008	1.970	0.160	3.267	0.071	0.480	0.489	2.218	0.136	0.130	0.719	0.140	0.708
hormone alone	18	0.591	0.442	0.226	0.635	0.069	0.793	0.071	0.790	1.303	0.254	0.961	0.327	0.050	0.823	0.184	0.668
hormone and chemo	16	2.613	0.106	3.937	0.047	0.531	0.466	1.071	0.301	0.707	0.401	1.778	0.182	0.317	0.573	0.101	0.751
hormone and radiation	10	1.833	0.176	2.709	0.100	0.837	0.360	0.987	0.320	2.049	0.152	2.485	0.115	0.075	0.784	0.011	0.915
hormone, chemo, radiation	21	0.703	0.402	0.601	0.438	1.036	0.309	1.139	0.286	0.065	0.799	0.240	0.624	0.047	0.829	0.089	0.765
chemo no (all subgroups)	30	0.833	0.361	3.472	0.062	0.403	0.526	1.123	0.289	0.067	0.795	0.465	0.495	0.003	0.955	0.049	0.825
chemo yes (all subgroups)	89	6.588	0.010	10.593	0.001	0.387	0.534	0.566	0.452	2.858	0.091	7.465	0.006	0.614	0.433	0.186	0.667
chemo (CMF)	55	5.455	0.020	9.345	0.002	1.275	0.259	1.561	0.212	1.522	0.217	4.847	0.028	0.152	0.697	0.016	0.899
chemo (AC/CMF)	34	1.289	0.256	1.608	0.205	0.420	0.517	0.317	0.574	1.300	0.254	2.740	0.098	0.507	0.477	0.456	0.500
chemo alone	13	1.865	0.172	1.268	0.260	0.107	0.743	0.245	0.620	0.322	0.570	1.356	0.244	0.099	0.753	0.126	0.722
chemo and radiation	39	4.123	0.042	8.229	0.004	0.898	0.344	0.209	0.648	2.380	0.123	3.271	0.071	0.431	0.512	0.180	0.672
radiation no (all subgroups)	47	1.964	0.161	3.638	0.057	0.009	0.927	0.067	0.796	0.005	0.946	0.497	0.481	1.310	0.252	1.819	0.178
radiation yes (all subgroups)	72	3.786	0.052	8.444	0.004	1.143	0.285	1.690	0.194	2.395	0.122	4.766	0.029	0.122	0.727	0.090	0.764

Logrank test and Wilcoxon test for the prediction of survival by the dichotomized immunohistochemical markers in the indicated subgroups of patients. Significant *p*-values are underlined

To assess whether the prediction of treatment responses can be strengthened when additional readouts are considered, we performed multivariate analysis. For survival with or without hormone therapy, exon 4 staining or osteopontin-c staining were analyzed as predictors in conjunction with other covariates (Table 7). Whereas osteopontin-c did not improve as a predictor, the combination of high exon 4 intensity plus high tumor grade worsened the prognosis without hormone therapy (exon 4 alone hazard ratio 0.503, *p*-value 0.016; with grade 0.450, 0.007), but maintains the favorable prognosis under treatment (exon 4 alone 0.657, 0.131; with grade 0.763, 0.352). Hazard ratios and *p*-values for survival under CMF chemotherapy showed improvement for the prediction with the combination of exon 4 intensity plus HER2 (0.398, 0.009), compared to exon 4 intensity alone (0.524, 0.019). Low HER2 and high staining intensity for osteopontin exon 4 increase the likelihood for resistance to CMF chemotherapy (Table 8).

**Table 7:**
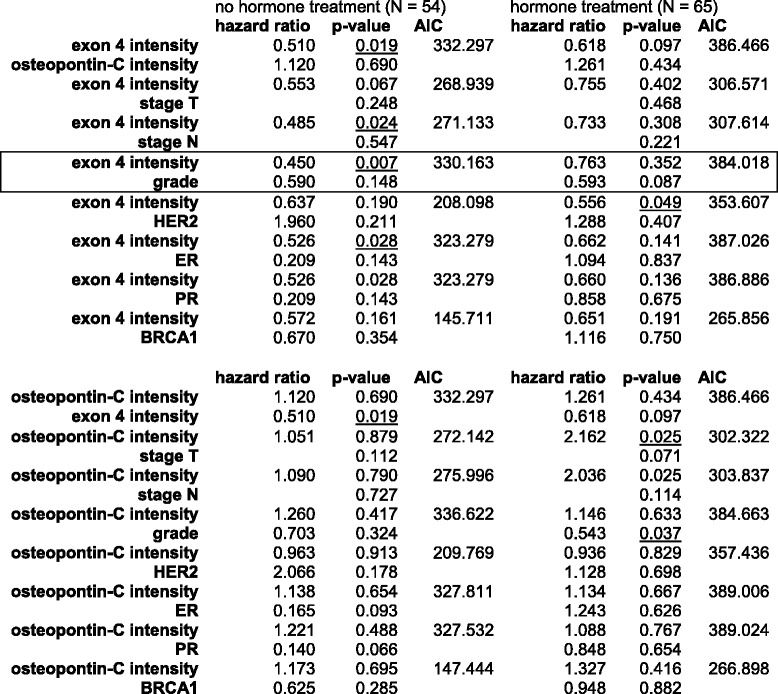
Multivariate analysis

Hazard ratios and *p*-values for survival under hormone therapy, using exon 4 staining (0–1 = low, 2–3 = high) or osteopontin-c staining (0–1 = low, 2–3 = high) plus other readouts as covariates. The combination of exon 4 intensity plus grade, which strengthens the prediction compared to exon 4 intensity alone, is boxed

**Table 8:**
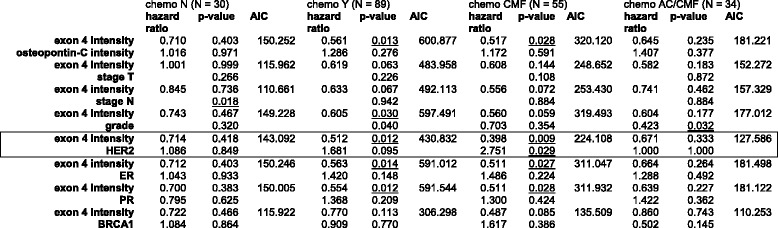
Multivariate analysis

Hazard ratios and *p*-values for survival under chemotherapy, using exon 4 staining (0–1 = low, 2–3 = high) plus other readouts as covariates. The combination of exon 4 intensity plus HER2, which strengthens the prediction compared to exon 4 intensity alone, is boxed. Tumor grade was included as low (grade 1) or high (grades 2–3). The parameters for tumor size and lymph node involvement, were not dichotomized and had different hazard ratios for each level (not shown). Significant *p*-values (*p* < 0.05) are underlined

## Discussion

In this study, we have identified osteopontin exon 4 as a predictor for a favorable treatment response to tamoxifen (the staining intensity is negatively correlated with survival in the non-hormone treated group, but not in the hormone treated group), but for resistance to chemotherapy with CMF (high level expressors have reduced survival in the treated group, but not in the non-treated group). The combination of high staining for osteopontin exon 4 with HER2 negative status appears to increase the likelihood for chemotherapy resistance. By contrast, osteopontin-c is prognostic, but may not be predictive for the therapeutic regimens applied here. Notably, while osteopontin-c is not a predictor of survival in the hormone treated group, a trend for it to be negatively associated with survival time in the non-hormone treated group fell just short of indicating significance by Cox hazard ratio (*p* = 0.096) and logrank test (*p* = 0.066), while significance was attained with the Wilcoxon test (*p* = 0.021). Nevertheless, in multivariate analysis no improvement was achieved for prediction with osteopontin-c. It is therefore more likely that a favorable response to hormone therapy is encoded in an osteopontin-a specific domain than in a common domain of osteopontin. The resistance to chemotherapy is selective to exon 4. As osteopontin-b is barely expressed in breast cancer and the protein is rapidly degraded [13], it is implied that osteopontin-a is the splice form responsible for resistance to chemotherapy.

The favorable response to anti-estrogens by breast cancers with high osteopontin-a levels may reflect the property of the osteopontin gene as a sentinel for estrogen responsiveness in mammary cells. Although an estrogen-response element is not present in the promoter, there are 7 steroid factor-response element-like sequences in this region. Expression may be induced by estrogen via ERRα in a context-dependent manner [14, 15]. Tumors with high levels of osteopontin-a probably are highly sensitive to estrogen signals (mediated through ER or through ERR), and therefore will be more likely to be susceptible to tamoxifen treatment. In immunohistochemistry, the abundance of osteopontin may be more accurately reflected in staining intensity than in percent positivity, because the percentage of area stained is much more susceptible to the placement of the section than is the intensity, making the former a weaker readout. In a related setting, for ER in breast cancer, it has been shown that the threshold of immunoreactivity is more important than the percentage positive in the generation of discordant or false-negative assays [16].

The abundance of exon 4 is predictive of resistance to chemotherapy with CMF, but not with AC/CMF. This was initially surprising as osteopontin was not previously known to convey resistance to methotrexate or fluorouracil. In contrast, the two AC agents, doxorubicin (adriamycin) and cyclophosphamide, have been reported to be subject to osteopontin-mediated drug resistance. The finding may point to translational limitations for testing drug sensitivity with mono-therapy in cell culture, as was done in the published resistance studies [1–4]. It may also be reflective of the benefit conveyed with altering drug combinations over treatment cycles, not only to alleviate toxicity but also to enhance efficacy. By broadening and alternating the drug regimen, the response to treatment in our breast cancer patients could be enhanced.

Discrepancies between this study and previous reports that had indicated an association between osteopontin levels and radiation resistance require an explanation. We see three likely causes. a) While the regimens tested here had overlap with the ones earlier associated with resistance, the treatments were complex and included components that may have helped to overcome resistance. Most patients in this study received radiation in conjunction with hormone therapy and/or chemotherapy. b) Overall, the efficacies of radiation treatment were low, reflected in poor survival in the treated groups. The underlying reason is that more aggressive treatment is given to patients with worse prognosis and could be interpreted to mean that all patients receiving radiation were in essence resistant. Therefore, no differences were discernible between the marker (osteopontin-c or exon 4) high and low groups. c) It is possible that the observations of this study may be compromised by its moderate power (119 patients). We previously reported that osteopontin-c is correlated to tumor grade [13] and the combination of both readouts slightly improves the prediction of survival [8]. In the present study, no correlation was identified between osteopontin-c and tumor grade or exon 4 and tumor grade, which may be attributable to the limited group size. The lack of significance for predicting survival by dichotomized osteopontin-c in the logrank test might seem to support this possibility.

Beside breast cancer, pan-osteopontin levels have been associated with treatment responses of prostate cancer to taxanes/androgen deprivation [17], of lung cancer (NSCLC) to carboplatin/paclitaxel [18], and of colorectal cancer to FOLFIRI-bevacizumab [19] (as osteopontin interacts with VEGF in multiple ways [20] it would be expected to affect the response to bevacizumab). In some of the predecessor studies, however, it has not been clear whether osteopontin was functionally linked to a reduced treatment response or independently predicted poor survival, because tumors with higher levels of the biomarker are more aggressive and inherently have a bad prognosis. A mechanistic connection of pan-osteopontin to drug resistance was established in prostate cancer. Osteopontin binds to integrin α_v_β_3_ and concentration- and time-dependently upregulates the efflux transporter ABCB1 (P-glycoprotein, PGP), which causes resistance to drugs that are substrates for this transporter [21]. Similarly in breast tumor cells, osteopontin may activate the hedgehog pathway and enhance drug resistance through GLI1-dependent regulation of ABCB1 and ABCG2 [4].

## Conclusions

In conclusion, immunohistochemistry of osteopontin splice variants has potential to aid decision making in breast cancer treatment. Specifically, osteopontin exon 4 is associated with a favorable response to tamoxifen, but a poor response to chemotherapy with CMF (cyclophosphamide, methotrexate, fluorouracil). While being prognostic, osteopontin-c may not be a significant predictor for sensitivity to treatment.

## Abbreviations

AC, cyclophosphamide, doxorubicin; AIC, Aikaike information criterion; CMF, cyclophosphamide, methotrexate, fluorouracil; ER, estrogen receptor; OPN, osteopontin; PR, progesterone receptor; sem, standard error of the mean; Std, standard deviation
